# Interaction of Gamma-Herpesvirus Genome Maintenance Proteins with Cellular Chromatin

**DOI:** 10.1371/journal.pone.0062783

**Published:** 2013-05-07

**Authors:** Nouman Mughal, Giuseppe Coppotelli, Simone Callegari, Stefano Gastaldello, Maria G. Masucci

**Affiliations:** Department of Cell and Molecular Biology, Karolinska Institutet, Stockholm, Sweden; Lisbon University, Portugal

## Abstract

The capacity of gamma-herpesviruses to establish lifelong infections is dependent on the expression of genome maintenance proteins (GMPs) that tether the viral episomes to cellular chromatin and allow their persistence in latently infected proliferating cells. Here we have characterized the chromatin interaction of GMPs encoded by viruses belonging to the genera Lymphocryptovirus (LCV) and Rhadinovirus (RHV). We found that, in addition to a similar diffuse nuclear localization and comparable detergent resistant interaction with chromatin in transfected cells, all GMPs shared the capacity to promote the decondensation of heterochromatin in the A03-1 reporter cell line. They differed, however, in their mobility measured by fluorescence recovery after photobleaching (FRAP), and in the capacity to recruit accessory molecules required for the chromatin remodeling function. While the AT-hook containing GMPs of LCVs were highly mobile, a great variability was observed among GMPs encoded by RHV, ranging from virtually immobile to significantly reduced mobility compared to LCV GMPs. Only the RHV GMPs recruited the bromo- and extra terminal domain (BET) proteins BRD2 and BRD4 to the site of chromatin remodeling. These findings suggest that differences in the mode of interaction with cellular chromatin may underlie different strategies adopted by these viruses for reprogramming of the host cells during latency.

## Introduction

A distinctive characteristic of herpesviruses is the capacity to establish lifelong infections where the virus persists in healthy carriers by hiding in a cellular reservoir that expresses only few latency-associated viral genes. Members of the lymphotropic gamma-herpesvirus subfamily establish latency in proliferating cells and have evolved specific strategies to avoid loss of the viral episomes during cell division. To this end, all gamma-herpesviruses express proteins, known as the Genome Maintenance Proteins (GMPs), that share two common characteristics: (i) they can initiate the replication of viral episomes in latently infected cells and coordinate the replication of the viral and cellular genomes during S-phase, and (ii) they act as bridges to tether the viral episomes to the host cell chromosomes during mitosis, ensuring thereby their correct partitioning between daughter cells [1]. These functions are dependent on protein domains that mediate the interaction with the origin of latent viral DNA replication, *OriP*, and with cellular chromatin, respectively.

The gamma-herpesviruses subfamily includes two major genera, the Lymphocryptoviruses (LCV) and Rhadinoviruses (RHV) of which the human tumor viruses Epstein-Barr virus (EBV) and Kaposi Sarcoma associated herpes virus (KSHV) are the best-characterized members, respectively [2]. The GMPs encoded by these viruses show very little sequence similarity, although members of the same genera show a higher degree of homology and similar protein domain organization. The highest degree of conservation is found within the C-terminus of all GMPs that bind to the viral *OriP*, whereas the N-terminus that anchors the viral episome to cellular chromatin is different in LCVs and RHVs. The EBV encoded GMP, EBNA1, binds to cellular chromatin via two N-terminal domains, known as the linking regions LR1 and LR2, that contain multiple repeats of the Arg-Gly-Arg motif that is found in many DNA and RNA binding proteins [3], [4]. The linking regions, hereafter indicated as GR1 and GR2, are separated by a Gly-Ala repeat (GAr) domain of variable length in different EBV isolates [5]. The GR1, GR2 and GAr domains are highly conserved in all LCVs although the viruses isolated from non-human primates often encode GMPs with short GAr that also contain interspersed Ser residues [6]. The GRs resemble the AT-hook of high mobility group A (HMGA) architectural proteins that bind to the minor groove of AT-rich DNA and promote chromatin remodeling with wide-ranging effects on cellular transcription, activation and differentiation [7]. While several lines of evidence suggest that the GRs may directly bind to cellular DNA [3], [8], [9], the tethering of EBNA1 to cellular chromatin in different phases of the cell cycle or nuclear sub-compartments may be facilitated by the interaction with several DNA binding proteins, such as the EBNA1-binding protein-2 (EBP2, [10]), high mobility group B protein -2 (HMGB2) [11] and others. The mode of interaction of RHV GMPs with cellular chromatin is less characterized. The N-terminal and C-terminal domains of these proteins are highly conserved [12], and the basic N-terminal domain of the KSHV encoded LANA1 was shown to interact with an acidic domain located at the interface between histone H2A and H2B on nucleosomes [13]. In addition, several cellular proteins have been implicated in the genome tethering mechanism of KSHV. Recruitment of LANA1 to mouse chromosomes was shown to require methyl CpG-binding protein (MeCP2) and DEK [14], which may facilitate nucleosome binding, while the C-terminal domain interacts with several chromatin-binding proteins including BRD4, BRD2/Ring3, Histone H1 and the nuclear mitotic apparatus protein NuMA [15], [16].

Although latently infected cells may carry several hundred copies of the viral episome, only a fraction of the GMP expressed in infected cells is likely to be required for anchoring the viral genomes to mitotic chromosomes. Thus, while the episome tethering and viral promoter trans-activating functions of the GMPs are relatively well understood, the overall effect of the interaction of these proteins with cellular chromatin is less clear. Indeed, EBNA1 was shown to bind to multiple sites on interphase chromatin [17] and ectopic expression is associated with a broad rearrangement of the cellular transcription profile [18]–[20]. The expression of LANA1 in KSHV negative cells was shown to induce an extensive reorganization of the cellular chromatin [21]. We have recently reported that, similar to HMGA proteins, EBNA1 is highly mobile and promotes chromatin decondensation in the A03-1 reporter cell line with slow kinetics and without recruitment of ATP-dependent chromatin remodelers [9]. This chromatin remodeling effect was dependent on the AT-hook-like domain and correlated with displacement of linker histone H1.

Here we have compared the chromatin interaction and chromatin remodeling capacity of GMPs encoded by LCVs and RHVs. We report that all tested GMPs were capable of inducing the decondensation of heterochromatin in A03-1 cells, but they differed in mobility measured by Fluorescence Recovery after Photobleaching (FRAP) and in the capacity to recruit accessory molecules required for the chromatin remodeling function.

## Materials and Methods

### Antibodies

Rabbit polyclonal anti-GFP antibody was purchased from Santa Cruz Biotechnology Inc. (Delaware Avenue, Santa Cruz, CA). The donkey horseradish peroxidase conjugated anti-rabbit IgG antibody was from GE Healthcare Bioscience (Pittsburgh, PA, USA).

### Plasmids

An overview of the nomenclature and source of the GMPs included in the study is shown in Table 1. The pFLAG-GFP-EBNA1 expression vector was kindly provided by E. Kieff (Harvard Medical School, Boston MA). The pFLAG-mCherry-Lac-EBNA1 (EBNA1) [9] and pSP64-papioEBNA1 (baEBNA1) [22] expression vectors were described previously. The baEBNA1 coding sequence was amplified from pSP64-papioEBNA1 with the primers pair: fw-AAAAAGCTTATGTCTGATGAGGGG CCTGGACCG, rev-AAAGCGGCCGCTCACTCCTGCCCTTCCTCACCCTC and cloned in the *HindIII/NotI* sites of p3xFLAG-CMV10. To generate p3xFLAG-CMV10.0-mCherry-LacR-baEBNA1, the mCherry-LacR coding sequence was excised from p3xFLAG-mCherry-LacR-HMGA1a [9] by *HindIII* digestion and the fragment was ligated in the *HindIII* site of pcDNA3-baEBNA1. To generate p3xFLAG-CMV10-GFP-baEBNA1, the GFP coding sequence was amplified from pEGFP-N1 (Clontech Laboratories Inc., Mountain View, CA, USA) with the primers pair: fw-AAAAAGCTTGGATCCATGGTGAGCAAGGGCGAGGAGC, rev-AAAGGATCCCT TGTACAGCTCGTCCATGCCGAG and cloned in the *BamHI* site of pcDNA3-baEBNA1. The p3xFLAG-CMV10-KSHV-LANA plasmid was a kind gift of E. Kashuba (Karolinska institute, Stockholm, Sweden). To generate p3xFLAG-CMV10-mCherry-LacR-LANA1, the mCherry-LacR coding sequence was amplified from p3xFLAG-mCherry-LacR-HMGA1a with the primers pair: fw-AAAAAGCTTAT GGTGAGCAAGGGCGAGGAG, rev-AGCTCGGTACCAAACCTTCCTCTTCTTCTT AGG and ligated in the *HindIII/KpnI* of p3xFLAG-CMV10-KSHV-LANA. To generate p3xFLAG-CMV10-GFP-LANA1, the GFP coding sequence was excised from p3xFLAG-GFP-HMGA1a by digestion with *HindIII* and the fragment was ligated into *HindIII* digested p3xFLAG-CMV10-KSHV-LANA. The p3xFLAG-CMV10-RFHV-LANA (mnR1-LANA1), p3xFLAG-CMV10-NRV-LANA (mnR2-LANA1) and p3xFLAG-CMV10-NRV-LANA-EGFP expression vectors were kindly provided by T.M. Rose (University of Washington, Seattle). To generate p3xFLAG-CMV10-mCherry-LacR-mnR1-LANA and p3xFLAG-CMV10-mCherry-LacR-mnR2-LANA1, the mCherry-LacR coding sequence was excised from p3xFLAG-mCherry-LacR-HMGA1a by digesting with *HindIII* and the fragment was ligated into *HindIII* digested p3xFLAG-CMV10-RFHV-LANA and p3xFLAG-CMV10-NRV-LANA. To generate p3xFLAG-CMV10-GFP-RFHV-LANA, the GFP coding sequence was excised from p3xFLAG-GFP-HMGA1a by digestion with *HindIII* and the fragment was ligated into *HindIII* digested pcDNA3-RFHVMnLANA. The p3xFLAG-CMV10-HVS orf73 plasmid was kindly provided by R.P. Searles (Oregon Health & Science University, West Campus, Beaverton, OR, USA). To produce p3xFLAG-mCherry-LacR-saLANA1, the mCherry-LacR coding sequence was amplified from p3xFLAG-mCherry-LacR-HMGA1a with the primers pair: fw-AAAGGTACCATGGTGAGCAAGGGCGAGGAG; rev-TTTGGTACCAACCTTCCTCTTCTTCTTAGG and cloned in the *KpnI* site of p3xFLAG-CMV10-HVS orf73. To produce p3xFLAG-CMV10-GFP-saLANA1, the GFP coding sequence was amplified from pEGFP-N1 with the primers pair: fw-AAAGGTACCATGGTGAGCAAGGGCGAGGAGC, rev-TTTGGTACCCTTGTAC AGCTCGTCCATGCCGAG and cloned in the *KpnI* site of p3xFLAG-CMV10-HVS orf73. The pVR1255-MHV-68 orf73 expression vector was kindly provided by J. Stewart (University of Liverpool, Liverpool, UK). To generate p3xFLAG-CMV10-muLANA1, the MHV-68 orf73 coding sequence was amplified from pVR1255-MHV-68 orf73 with the primers pair: fw-AAACTCGAGATGCCCACATCCCCACCGACTACA, rev-AAAGCGGCCGCTTATGTCTGAGACCCTTGTCCCTGT and cloned into the *XhoI/NotI* sites of the p3xFLAG-CMV10 plasmid. To generate p3xFLAG-CMV10-mCherry-LacR-muLANA1, the mCherry-LacR coding sequence was amplified from p3xFLAG-mCherry-LacR-HMGA1a with the primers pair: fw-AAACTCGAGATGGT GAGCAAGGGCGAGGAG; rev-AAACTCGAGAACCTTCCT CTTCTTCTTAGG and clones in the *XhoI* site of p3xFLAG-CMV10-muLANA1. To generate p3xFLAG-CMV10-GFP-muLANA1, GFP was amplified from pEGFP-N1 with the primers pair: fw-AAACTCGAGATGGTGAGCAAGGGCGAGGAGC, rev-AAACTCGAGCTTGTA CAGCTCGTCCATGCCGAG and cloned in the *XhoI* site of p3xFLAG-CMV10-muLANA1. The mCherry-LacR [23], mCherry-LacR-NLS-VP16 [9], GFP-NLS and YFP-BRG1 [24] and GFP-CHD4 [25] plasmids were described previously. SNF2H-GFP was kindly provided by H. van Attikum (Leiden University Medical Center, Leiden, Netherland) and plasmids expressing YFP tagged GCN5, CAF, P300, Brd2 and Brd4 were kindly provided by I. Rafalska-Metcalf (The Wistar Institute, Philadelphia, Pennsylvania, USA). The identity of all plasmids was confirmed by sequencing.

**Table 1:** Gamma-herpesvirus GMPs included in this study.

Species	Acronym	Genus	Common name	GMP	UniProtAccession	Ref
Human herpesvirus 4	HHV-4	LCV^a^	Epstein-Barr virus (EBV)	EBNA1	P03211	[39]
Cercopithecine herpesvirus 12	CeHV-12	LCV	Baboon herpesvirus; Papiine herpesvirus 1;Herpesvirus papio (HVP)	baEBNA1	Q80890	[40]
Human herpesvirus 8	HHV-8	RHV^b^	Kaposi's sarcoma-associated herpesvirus(KSHV)	LANA1	E5LC01	[41]
Macaca nemestrina rhadinovirus 2	MneRV2	RHV	Macaca nemestrina rhadinovirus 2; (NRV)	mnR2-LANA	A1XYW0	[12]
Cercopithecine herpesvirus 18	CeHV-18	RHV	Macaca nemestrina rhadinovirus 1;Retroperitoneal fibromatosis associatedherpesvirus (RFHV)	mnR1-LANA	A1XYV9	[12]
Saimirine herpesvirus 2	SaHV-2	RHV	Herpesvirus saimiri 2; Herpesvirus saimiri (HVS)	saLANA	Q80AG4	[42]
Murid herpesvirus 4	MuHV-4	RHV	Murine herpesvirus 68 (MHV68)	muLANA	O41974	[43]

a. LCV = Genus Lymphocryptovirus.

b. RHV = Genus Rhadinovirus.

### Cell Lines and Transfection

The human osteosarcoma line U2OS, cervical carcinoma cell line HeLa and murine fibroblast NIH3T3 were grown in Iscove’s medium supplemented with 10% Fetal Bovine Serum (FBS) and 10 µg/ml Ciprofloxacin, all from Sigma-Aldrich (St. Louis, MO, USA). The Chinese Hamster Ovary carcinoma cell line A03-1 that harbors multi-copies of the 256×LacO array [26] was grown in DMEM/F12 complete medium (Life Technologies, Grand Island, NY, USA). All cells were kept at 37°C in a 5% CO_2_ incubator. For transfection, semi-confluent monolayers were grown on ∅22 mm or ∅42 mm cover slides **(**VWR International®, Radnor PA, USA) placed in 6 wells plate or in 6 cm dishes and transfected with 1.0 µg of the indicated plasmid using the jetPEI® transfection kit (Polyplus Transfection, Illkirch, France).

### Western Blot

Cell lysates were prepared in RIPA buffer (150 mM TRIS-HCl pH 7.5, 150 mM NaCl, 2 mM EDTA, 1% Triton, 1.0% SDS and protease inhibitor) and protein concentration was measured using the BIO-RAD DC Protein Assay (BIO-RAD Laboratories, Inc. Hercules, CA). Twenty µg of proteins were fractionated in NUPAGE® Novex® Bis-tris precast polyacrylamide gels (Life Technologies, Grand Island, NY, USA) and blotted on 0.45 µm Immobilon™ PVDF membrane (MILLIPORE Corporation, Bedford, MA USA). After incubation with the indicated primary and secondary antibodies, the immune complexes were detected by chemiluminescence (Thermo Scientific Pierce ECL, Rockford, IL, USA).

### Fluorescence Analysis

Cells expressing GFP, YFP or mCherry tagged proteins grown on glass cover slides were rinsed with Phosphate Buffer Saline (PBS), fixed with 4% formaldehyde in PBS for 15 min and permeabilized with 0.5% Triton X-100 for 20 minutes before mounting with VECTASHIELD medium containing DAPI (Vector Laboratories Inc. Burlingame, CA). Where indicated, detergent extraction was performed before fixation by incubating the slides for 5 min on ice in buffer containing 137 mM NaCl, 20 mM D-glucose, 20 mM HEPES, 5.4 mM KCl, 1.8 mM CaCl_2_, 0.8 mM MgSO_4_ and 0.5% Triton-X100, pH 7. Digital images were captured with a Zeiss LSM510 META Confocal microscope and analyzed with the ImageJ 1.42 q software (Wayne Rasband, National Institutes of Health, USA).

### Nucleosome Array Conformation Analysis

A03-1 cells were seeded on ∅22 mm cover slides placed in 6 wells plate and when 60% confluent transfected with the indicated plasmid. After 48 hours, the slides were fixed, permeabilized and mounted with VECTASHIELD medium containing DAPI (Vector Laboratories, Inc. Burlingame, CA). Digital images were captured with a Zeiss LSM510 META Confocal microscope and analyzed with the ImageJ software (Rasband, W.S., U. S. National Institutes of Health, Bethesda, Maryland, USA). The relative size of the array is expressed as percentage of the nuclear area.

### Fluorescence Recovery After photobleaching (FRAP)

FRAP assays were performed in transiently transfected U2OS cells with a Zeiss LSM 510 META confocal microscope equipped with a CO_2_ chamber. For protein mobility analysis 30 pre-bleach images were acquired in a strip region of 1.74×17.86 µm using a Plan-Apochromat 63x/1.4 Oil Dic objective zoom 8. After a bleach pulse of 5 iterations, images were acquired with a scan speed of 38.4 msec/cycle and a delay of 5 msec (600 images in 25.5 sec). The power of a 25-mW argon laser (488-nm line) was set to 0.1% for imaging and 100% for bleaching. FRAP recovery curves were generated by normalizing the fluorescence intensity in the bleached area to the initial fluorescence in the same area. In a typical experiment 10 cells were analyzed and each experiment was repeated at least three times. In order to compare the recovery curves of different proteins and correct for fluorescence bleaching during acquisition, the curves were normalized relative to the fluorescence at the time of bleaching (Fx(t0)  = 0% recovery) and the fluorescence of fully recovered GFP-NLS (F_GFP-NLS_ (t-end)  = 100% recovery) using the formula: Fx’(tn) = ^hello^Fx(tn)-Fx(t0)]/^hello^F_GFP-NLS_(t-end)-F(t0)] where t-end is 25.5 sec. The mean ± SD of the % recovery achieved at T-end was calculated for each protein.

### Sequence Analysis

The amino acid sequences of the B95.8 EBNA1 (P03211), baEBNA1 (Q80890), KSHV LANA1 (E5LC01), mnR1-LANA (AIXYV9), mnR2-LANA (AIXYW0), saLANA (Q80AG4) and muLANA (O41974) were retrieved from the UniProt database (http://www.uniprot.org, release 2012–11). Multiple sequence alignments were generated with CLUSTAL-W [27] in order to identify functional conserved domains. JALVIEW Version 2-a [28] and DOG 2.0 [29] were used for graphical output of the sequence alignments and schematic illustration of the domains.

### Statistical Analysis

Statistical analysis was performed with the Prism5 software (GraphPad Software Inc., La Jolla CA, USA). Significant probabilities are indicated as: p<0.05(*), p<0.01 (**) and p<0.001 (***).

## Results

### The GMPs Show a Diffuse Localization in Interphase Nuclei and do not Accumulate on Heterochromatin

**Figure 1:**
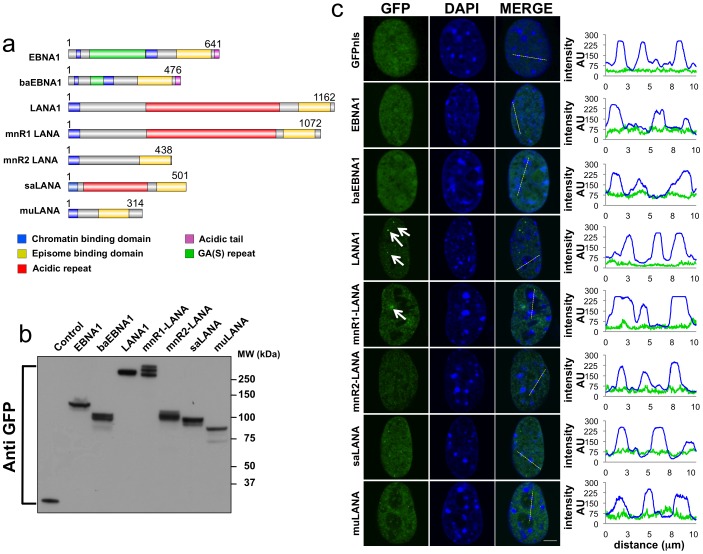
Localization of the GMPs in interphase nuclei. Schematic illustration of the domain organization of the GMPs included in the study. For nomenclature and references see Table 1. (a) Protein domains were identified as regions of high homology using Clustal W multiple sequence alignments. The C-terminal episome-binding domains are indicated in yellow and the chromatin targeting modules in blue. The Gly-Ala or Gly-Ala-Ser repeats of LCV-GMPs and the acidic repeats of RHV-GMPs are indicated in green and red, respectively. The acidic tails of LCV-GMPs are shown in pink. (b) Expression of GFP tagged GMPs in transfected U2OS cells. Representative western blot of total lysates from U2OS cells harvested 48 hrs after transfection probed with an anti-GFP antibody. (c) Nuclear localization of the transfected proteins. Representative fluorescence micrographs of NIH3T3 cells transfected with the indicated GFP-GMP fusion proteins or control GFP-NLS. DNA is visualized by DAPI staining (blue). The merged image and localization profile indicate that the GMPs do not accumulate on heterochromatin.

In order to characterize the chromatin binding properties of GMPs encoded by different LCVs and RHVs, we collected a representative panel of GFP-tagged recombinant proteins (Table 1). The domain organization of the GMPs included in the study is illustrated in Figure 1a. All GMPs contain relatively conserved C-terminal viral episome-binding domains (Figure 1a, yellow boxes), whereas the N-terminal chromatin targeting modules (Figure 1a, blue boxes) differ among the families. The N-terminus of LCV GMPs contains multiple Arg-Gly-Arg repeats that resemble the AT-hook of HMGA proteins [3], whereas a basic N-terminal domain is likely to be involved in the interaction of RHV GMPs with nucleosomes or nucleosome binding proteins [30]. In addition, the LCV GMPs contain internal repetitive sequences of Gly-Ala or Gly-Ala-Ser of different length (Figure 1a, green boxes), and a C-terminal acidic tails (Figure 1a, pink boxes), whereas some but not all RHV GMPs contain internal acidic repeats of different length and amino acid composition (Figure 1a, red boxes).

Analysis of protein expression in western blots of transfected U2OS cells probed with an anti-GFP antibody confirmed the expression of polypeptides of the correct size in all cases except for mnR2-LANA that migrated approximately 20 kD above the expected size (Figure 1b). Since the length of the coding sequence and correct reading frame were confirmed by restriction nuclease digestion and sequencing, the higher molecular weight is likely to be due to extensive post-translational modification. The subcellular localization of the GFP-tagged proteins was compared by fluorescence microscopy in transfected mouse NIH3T3 cells (Figure 1c). This cell line was chosen due to the easy visualization of heterochromatic regions in the nuclei of murine cells stained with DAPI. As expected, all proteins showed an exclusive nuclear localization and exhibited a diffuse fluorescence with no apparent association with distinct nuclear sub-compartments. In accordance with previous reports [31], bright nuclear speckles (indicated by arrows in Figure 1c) were observed in LANA1 expressing cells, and a similar pattern was also detected in cells expressing mnR1-LANA and saLANA, although the size of the speckles varied significantly in different transfection experiments. Analysis of the localization profile of green (GFP-tagged GMPs) and blue (DAPI stained DNA) fluorescence revealed that the GMPs did not accumulate on heterochromatin that appears as bright dots in the blue fluorescence channel (Figure 1c, right panels).

### The GMPs Interact with Chromatin with Similar Avidity

**Figure 2:**
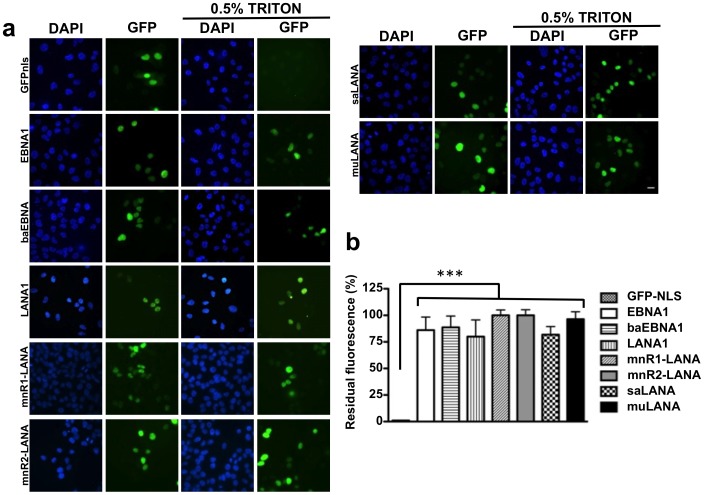
Detergent resistant interaction of the GMPs with cellular chromatin. Representative images illustrating the nuclear fluorescence of U2OS cells transiently expressing the indicted GFP-tagged GMPs without or with treatment for 5 min in the presence of 0.5% Triton X-100 before fixation. (a) The nuclei are visualized by staining with DAPI. Digital images were captured using a LEITZ-DMRB fluorescence microscope equipped with a CCD camera. (b) The fluorescence intensity was quantified in 100 nuclei from each condition using the ImageJ software. The percentage of residual fluorescence was calculated as (mean fluorescence treated cells/mean fluorescence untreated cells) ×100. The mean ± SE of three experiments is shown in the figure. *** = p<0.001.

In view of the different chromatin targeting modules of LCV and RHV encoded GMPs, we then asked whether this might influence the strength of interaction with cellular chromatin. We have previously shown that the interaction of EBNA1 with cellular chromatin is resistant to extraction with 0.5% Triton X-100, which prevents proteasomal degradation and contributes to the very slow turnover of the protein [8]. In order to test whether this property is shared by other GMPs, aliquots of U2OS cells were seeded on cover slides 24 hrs after transfection and the fluorescence intensity of cells grown for one more day was quantified with or without prior extraction with 0.5% Triton X-100 for 5 minutes (Figure 2a). As expected, the treatment resulted in loss of fluorescence in cells expressing GFP-NLS that does not interact with chromatin, whereas cells expressing the GFP-GMPs retained between 75% and up to 100% of the fluorescence detected in untreated cells (Figure 2b). Thus, all GMPs establish strong interactions with cellular chromatin.

### All GMPs Promote Chromatin Decompaction

**Figure 3:**
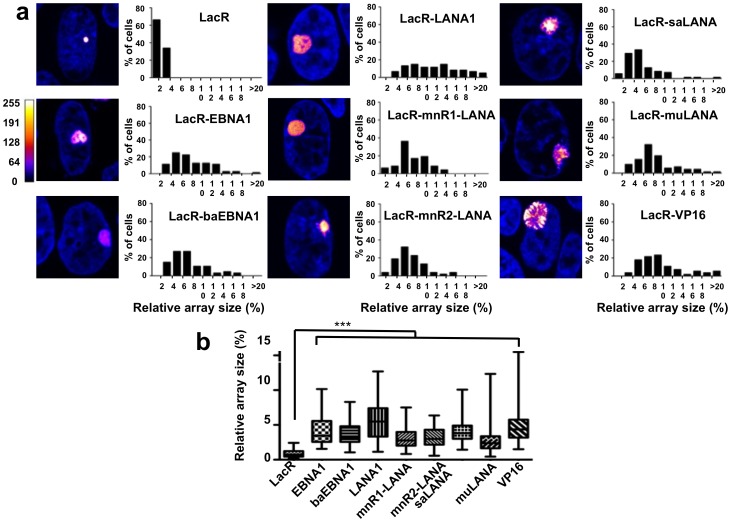
All GMPs promote chromatin decompaction. Representative confocal images illustrating the size of the LacO chromatin array in cells expressing LacR fused to the indicated GMPs. (a) A03-1 cells were transfected with plasmids encoding mCherry-LacR alone or the indicated fusion proteins and the size of the LacO array was measured 48 hrs after transfection. The LUT for the color-coding is shown on the left. Scale bar  = 2 µm. The size of the array relative to the size of the nucleus was calculated according to the formula: (area of the array/area of the nucleus x100) in more than 70 cells for each condition. The arrays size distribution is shown in the histograms on the right of the images. (b) Box plot illustrating the median and interquantile distribution of the size of the fluorescent arrays in cells expressing the different GMPs. The effect of the GMPs was comparable to that induced by the prototype viral transcativator VP16. *** = p<0.001.

Ectopic expression of EBNA1 and LANA1 is associated with global changes in the nuclear architecture [9], [21], suggesting that the GMPs may alter the organization of cellular chromatin. To address this possibility we took advantage of the A03-1 reporter cell line that carries multiple copies of a 256 repeat array of the *Lac operator* sequence (LacO) integrated in a 90 Mb heterochromatin region [26]. The heterochromatic area appears as an intensely fluorescent dot in cells expressing a chimeric Lac repressor (LacR) fused to mCherry (Figure 3a, upper panel). Targeting of chromatin remodelers to the array by fusion to LacR is accompanied by large-scale chromatin de-condensation that can be quantified as increased size of the fluorescent area relative to the size of the nucleus. Quantitative analysis of the size of the LacO array in A03-1 cells expressing LacR alone showed that the array occupied ≈2% of the nuclear area. In accordance with previous reports [32], extensive unfolding of the array was observed in the majority of the cells when the prototype viral transcativator encoded by the herpes simplex VP16 was targeted to the region by fusion to LacR (Figure 3a). By compiling the results obtained in three independent experiments where at least 50 cells were analyzed, we found that in mCherry-LacR-VP16 expressing cells the array occupied between 4% and up to 20% of the nuclear area, which corresponds to an average increase of 10 fold (Figure 3b). In spite of individual variations in the magnitude of the effect, targeting of the GMPs to the region by fusion to mCherry-LacR was in all cases accompanied by a highly significant increase in the average size of the array (Figure 3b). Interestingly, the effect was more pronounced in cells expressing the GMPs encoded by the human tumor viruses EBV and KSHV, EBNA1 and LANA1 respectively, where the array reached 20% of the nuclear area in a sizable proportion of the cells.

### LCV-GMPs and RHV-GMPs Show Different Mobility on Interphase Chromatin

**Figure 4:**
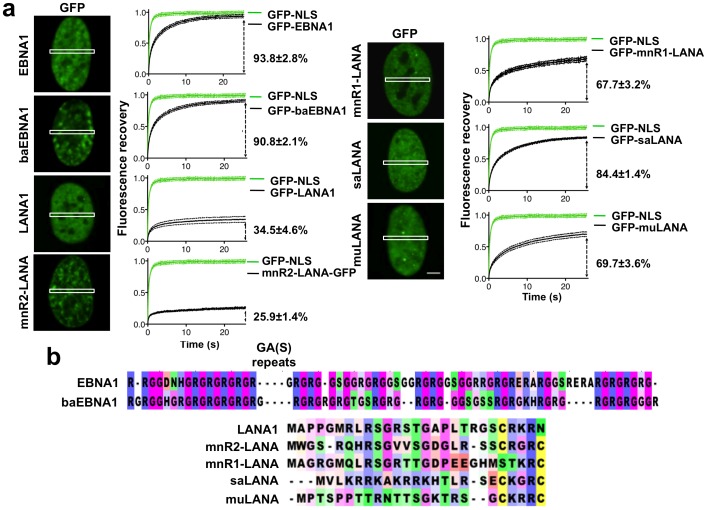
LCV- and RHV-GMPs show different mobility on chromatin. The mobility of GFP-GMP fusion proteins on interphase chromatin was assayed by FRAP in transiently transfected U2OS cells. (a) The normalized % Fluorescence recovery was calculated relative to the mean fluorescence at the time of bleaching (0% recovery) and the mean fluorescence of GFP-NLS after recovery for 25.5 sec (100% recovery). The mean ±SD of the relative fluorescence in 20 cells is shown in each graph. (b) Clustal W multiple sequence alignment of the chromatin targeting modules of LVC and RHV GMPs. LCV-GMP share highly conserved Gly-Arg repeat regions that resemble the AT-hook of HMGA proteins. LANA1, mnR1-LANA and mnR2-LANA share a highly conserved N-terminal domain that was shown to mediate the interaction of LANA1 with histone H2A–H2B. The chromatin binding domains of saLANA and muLANA have not been characterized.

In order to reveal features of the interaction of GMPs with cellular DNA that may be relevant for the chromatin remodeling function, the mobility of GFP-tagged proteins was measured in the nucleus of transiently transfected U2OS cells by fluorescence recovery after photobleaching (FRAP). The efficiency of FRAP recovery of the GFP chimeras were compared to that of a control nuclear GFP (GFP-NLS) that does not bind to chromatin. In accordance with previous reports [9], [11], EBNA1 was highly mobile on chromatin with a virtually full recovery of fluorescence by the end of the observation period of 25.5 sec (Figure 4a). A comparable virtually full recovery was observed with baEBNA1 that shares 56% amino acid sequence homology with EBNA1 and a highly conserved AT-hook-like chromatin targeting module (Figure 4b). In contrast, a poorer recovery was observed in cells expressing the RHV GMPs. Only 25% to 35% of the initial fluorescence was recovered in cells expressing LANA1 and mnR2-LANA within the observation period, suggesting a very slow overall mobility and possibly the presence of a large immobile fraction (Figure 4a). The two proteins belong to different RHV subfamilies but share a highly conserved domain structure (Figure 1a), and more than 45% sequence identity in the N-terminal domain that was shown to mediate the interaction of LANA1 with cellular chromatin via binding to histone H2A and H2B [13] (Figure 4b). The remaining RHV GMPs showed intermediate recoveries of 67.7%, 69.7% and 84.4% at the end of the observation period for mnR1-LANA, muLANA and, saLANA, respectively (Figure 4a). The N-terminal domain of nmR1-LANA is very similar to that of LANA1 and mnR2-LANA (Figure 4b), suggesting that they could have similar interacting partners, whereas the N-terminal domains of saLANA and muLANA share the overall prevalence of basic amino acid residues but no sequence similarity with the corresponding regions of other RHV GMPs.

### RHV GMPs Recruit BRD2 and BRD4 to the Site of Chromatin Remodeling

**Figure 5:**
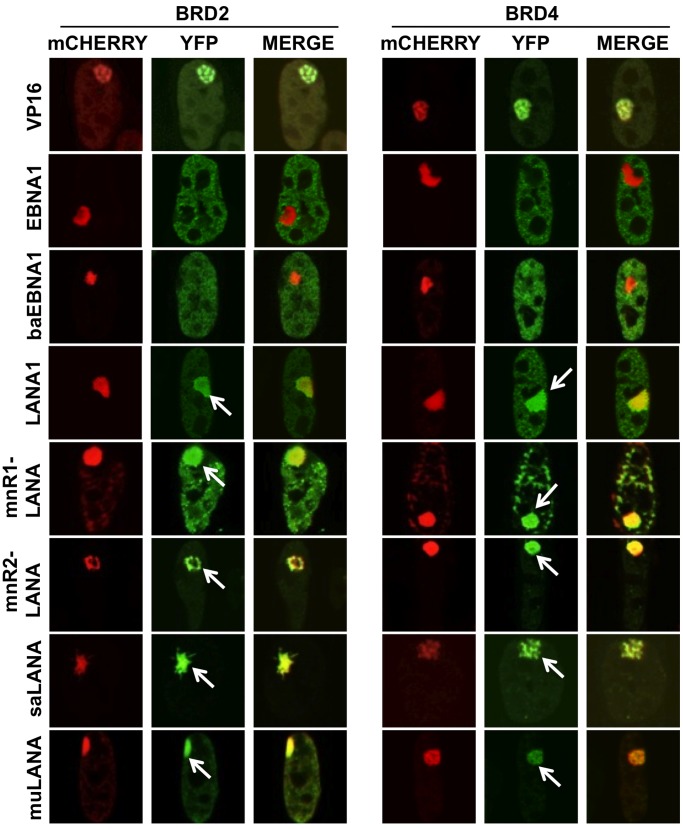
RHV-GMPs recruit BRD2 and BRD4 to the site of chromatin remodeling. Representative confocal images illustrating the nuclear fluorescence of A03-1 cells co-transfected with plasmids expressing mCherry-LacR-VP16 or mCherry-LacR-GMP fusion proteins and YFP-tagged BRD2 or BRD4. The recruitment of BRD2 and BRD4 to the site of chromatin decondensation, visualized by the accumulation of green fluorescence overlapping with the red fluorescent array, is indicated by arrows in cells expressing VP16 or the RHV-GMPs.

Powerful transcriptional transactivators, such as VP16, promote chromatin decondensation via recruitment of histone acetyltransferases and ATP-dependent chromatin remodelers whereas members of the HMG protein family initiate the remodeling process by competitive displacement of linker histones. We have previously shown that, similar to HMGAs, EBNA1 promotes chromatin remodeling with a slow kinetics and without recruitments of ATP-dependent remodeling complexes [9]. We now tested whether these properties are shared by other GMPs. To this end, we compared mCherry-LacR-tagged VP16 and GMPs for their ability to recruit a panel of GFP- or YFP-tagged ATPase subunits of known ATP-dependent remodeling complexes, including the BRG1 subunit of SWI/SNF complexes, the SNF2H subunit of ISWI complexes and the CHD4 subunit of NuRD complexes, histone acetyltransferases, such as GCN5, pCAF and p300, and two bromo and extra terminal domain (BET) proteins, BRD2 and BRD4, that bind to acetylated histones and are often hijacked by viruses to promote transcription [33]. As illustrated by the representative micrographs shown in Figure 5, Figure S1, Figure S2, and summarized in Table 2, all the tested proteins were recruited to the site of chromatin decondensation in cells expressing mCherry-LacR-VP16, as expected. In accordance with our previous findings, none of the proteins was recruited by EBNA1 and a similar behavior was observed with baEBNA1 that shares with EBNA1 a highly conserved AT-hook like chromatin-targeting module. In line with the presence of high affinity binding sites for BRD2 and BRD4 in the C-terminal domain of LANA1 [15], the two BET proteins were recruited to the site of chromatin remodeling in cells expressing mCherry-LacR-LANA1, and a similar recruitment was observed with all the RHV encoded GMPs (Figure 5 and Table 2). Recruitment of other components of ATP-dependent chromatin complexes and histone acetyltransferases was occasionally observed with different RHV GMPs (Figure S1, Figure S2 and Table 2). Thus, in some of the cells LANA1 recruited BRG1 and p300, and mnR1-LANA recruited pCAF, p300 and GCN5. These differences were highly reproducible in at least three independent experiments performed with each test combination, and could not be attributed to variations in the relative expression of the GMPs or the co-transfected cellular proteins.

**Table 2:** Recruitment of components of chromatin remodeling complexes by herpesvirus GMPs^a^.

	BRG1	SNF2H	CHD4	pCAF	p300	GCN5	BRD2	BRD4
**VP16**	**+**	**+**	**+**	**+**	**+**	**+**	**+**	**+**
**EBNA1**	**−**	**−**	**−**	**−**	**−**	**−**	**−**	**−**
**αbαEBNA1**	**−**	**−**	**−**	**−**	**−**	**−**	**−**	**−**
**LANA1**	**±**	**−**	**−**	**−**	**±**	**−**	**+**	**+**
**mnR1-LANA**	**−**	**−**	**−**	**±**	**±**	**±**	**+**	**+**
**mnR2-LANA**	**−**	**−**	**−**	**±**	**−**	**−**	**+**	**+**
**saLANA**	**−**	**−**	**−**	**−**	**−**	**−**	**+**	**+**
**muLANA**	**−**	**−**	**−**	**−**	**−**	**−**	**+**	**+**

a. More than 20 cells were examined in each test combination in three independent experiments. Score: **−** = recruitment to the array never observed; + =  clear recruitment to the array in all images; ± = recruitment to the array observed in some but not all images.

## Discussion

Intense studies have been devoted to the functional characterization of the GMPs encoded by gamma-herpesviruses with the hope that a better understanding of their properties may lead to the design of novel and highly specific antiviral therapeutics capable of halting the risk of malignant transformation. However, these studies have often overlooked an important aspect of the biology of these proteins, namely their capacity to interact with cellular chromatin throughout the cell cycle and independent of the viral genome tethering function, which could have important consequences for the host cells. In this study we have explored this issue by investigating the interaction with cellular chromatin of GMPs encoded by viruses belonging to the LCV and RHVs genera. We reasoned that the different chromatin-targeting modules of these GMPs might mediate different types of interaction with chromatin, which could differently affect the host cells. Our experimental approach based on the ectopic expression of GFP or otherwise tagged proteins in reporter cells lines suffers from a number of technical limitations, not least the overexpression of the transfected proteins outside of the natural context of infection. Nevertheless, the results provide interesting insights on the capacity of the GMPs to participate in the reprogramming of their host-cells by promoting a global remodeling of chromatin architecture.

We have found that GMPs encoded by LCVs and RHVs share the capacity to establish detergent-resistant interactions with cellular DNA and to promote the decompaction of heterochromatin in the A03-1 reported cell lines. The effect on heterochromatin unfolding is in line with the failure of the transfected GMPs to accumulate on heterochromatin (Figure 1c), and with the previously reported capacity of EBNA1 and LANA1 to release the bulk of DNA from heterochromatic foci and to promote extensive change in the positioning of chromosomes in interphase nuclei [9], [21]. The magnitude of the effects observed in our experiments was similar for all GMPs and comparable to that induced by the prototype viral transactivator HSV VP16 that was tested in parallel. Chromatin architecture plays a key role in the control of gene expression by regulating the access of transcription factors to their binding sites on DNA. Many transcription factors cooperate with activators that promote chromatin decondensation through recruitment of ATP-dependent remodeling complexes and post-translational modification of histone tails [34], [35]. In addition, both local and widespread effects on the accessibility of chromatin are induced by “architectural factors”, such as members of the three families of High Mobility Group (HMG) non-histone proteins, that bind in a sequence-independent manner to specific structures in DNA and cooperatively displace linker histones, leading to a local opening of chromatin and initiation of the gene activation process [36]. It is noteworthy that chromatin decompaction may allow the recruitment of both transcriptional activators and repressors. Thus, the effects on transcriptions may change depending on the cells type and the initial state of differentiation or activation. Our findings point to chromatin remodeling as an important shared feature of the GMPs, which could play an important role in the capacity of these proteins to regulate latent infection and, in cooperation with viral or cellular oncogenes, contribute to malignant transformation.

An important aspect of our work is the demonstration that, although the effect on chromatin organization appears to be similar, the interaction of the GMPs with cellular chromatin is profoundly different. We have found that two GMPs encoded by the EBV and HVP LCVs are highly mobile on chromatin and promote chromatin decompaction without recruitment of ATP-dependent remodeling complexes. This finding is in line with the highly conserved chromatin-targeting module of these proteins that resembles the AT-hook of HMGA architectural transcription factors and chromatin remodelers. In contrast, five RHV encoded GMPs varied considerably in their mobility, ranging from a significantly reduced mobility compared to LCV GMPs, to apparently immobile. Furthermore, their capacity to promote chromatin decompaction correlated with regular recruitment of the BET proteins BRD2 and BRD4 and, in some cases, other components of ATP-dependent chromatin remodeling complexes.

The consistent behavior of LCV and RHV GMPs suggests that their different chromatin-targeting modules could be a key determinant of mobility. Most importantly, while the LCV GMPs may directly bind to cellular DNA via the AT-hook, the targeting of RHV GMPs is achieved via interaction with DNA-binding proteins that may dictate the mobility of the complex. It is noteworthy that LANA1 binds to DNA via interaction with the nucleosome resident proteins histones H2A and H2B. In this context it was surprising to find a more efficient fluorescence recovery in mnR1-LANA compared LANA1 and mnR2-LANA (Figure 4a). The N-terminal of the three proteins is highly conserved with more than 45% sequence identity and one may therefore predict a similar mode of binding to the nucleosome. However, mnR1-LANA contains two acidic residues immediately adjacent to the basic surface involved in histone binding (Figure 4b), which may destabilize the binding and explain the increased mobility of this protein. The chromatin targeting modules of saLANA and muLANA have not been characterized but their N-terminus shares the overall basic charge observed in LANA1. Interestingly, the N-terminal domains of all RHV GMPs also contain several relatively well conserved Thr and Ser residues that are phosphorylated in LANA1 by the CK1, PIM1, GSK-3 and RSK3 kinases [37]. Short-term treatment of transfected cells with RSK inhibitors reduced the interaction of LANA1 with histone H2B and promoted protein degradation, suggesting a possible strategy for interfering with the binding of RHV GMPs to cellular chromatin.

The capacity of all RHV GMPs to recruit BRD2 and BRD4 to the site of chromatin remodeling is in agreement with the presence of multiple conserved high-avidity binding sites in the C-terminus of the molecules (reviewed in [33]). BRD2, BRD4 and related bromodomain proteins provide a scaffold on chromatin for the recruitment of E2F transcription factors, histone deacetylases, histone H4-specific acetyltransferase and protein complexes involved in chromatin remodeling, including SWI/SNF and elements of the Mediator complex [38]. While a precise dissection of the role of BRD2 and BRD4 in chromatin remodeling induced by RHVs requires further investigation, it is tempting to speculate that the high avidity interaction with GMPs could jump-start the process by bypassing the need for prior histone tail acetylation. This scenario may also explain the irregular recruitment of ATPases and HATs to the site of chromatin remodeling induced by the RHV GMPs, as opposed to the consistent recruitment observed in VP16 expressing cells, since indirect recruitment via binding to the BET proteins could result in weaker interactions that may escape detection in the experimental conditions of the assay.

## Supporting Information

Figure S1 **Recruitment of components of chromatin remodeling complexes by LCV and RHV GMPs.** Representative confocal images illustrating the nuclear fluorescence of A03-1 cells co-transfected with plasmids expressing the mCherry-LacR-VP16 or mCherry-LacR-GMP fusion proteins and the GFP-tagged ATPase subunits of the SWI/SNF, ISWI and CHD4 chromatin remodeling complexes, BRG1, SNF2H and CHD4, respectively. Recruitment to the site of chromatin decondensation, visualized by the accumulation of green fluorescence overlapping with the red fluorescent array, is indicated by arrows.
(TIFF)

Figure S2 **Recruitment of components of chromatin remodeling complexes by LCV and RHV GMPs.** Representative confocal images illustrating the nuclear fluorescence of A03-1 cells co-transfected with plasmids expressing the mCherry-LacR-VP16 or mCherry-LacR-GMP fusion proteins and the YFP-tagged acetyltransferases pCAF, p300 and GCN5. Recruitment to the site of chromatin decondensation, visualized by the accumulation of green fluorescence overlapping with the red fluorescent array, is indicated by arrows.
(TIFF)
