# Supplementary figures and images for: Interaction of Gamma-Herpesvirus Genome Maintenance Proteins with Cellular Chromatin
Source: PLoS One. 2013 May 7;8(5):e62783. doi: 10.1371/journal.pone.0062783 (PMC3646995; doi:10.1371/journal.pone.0062783)

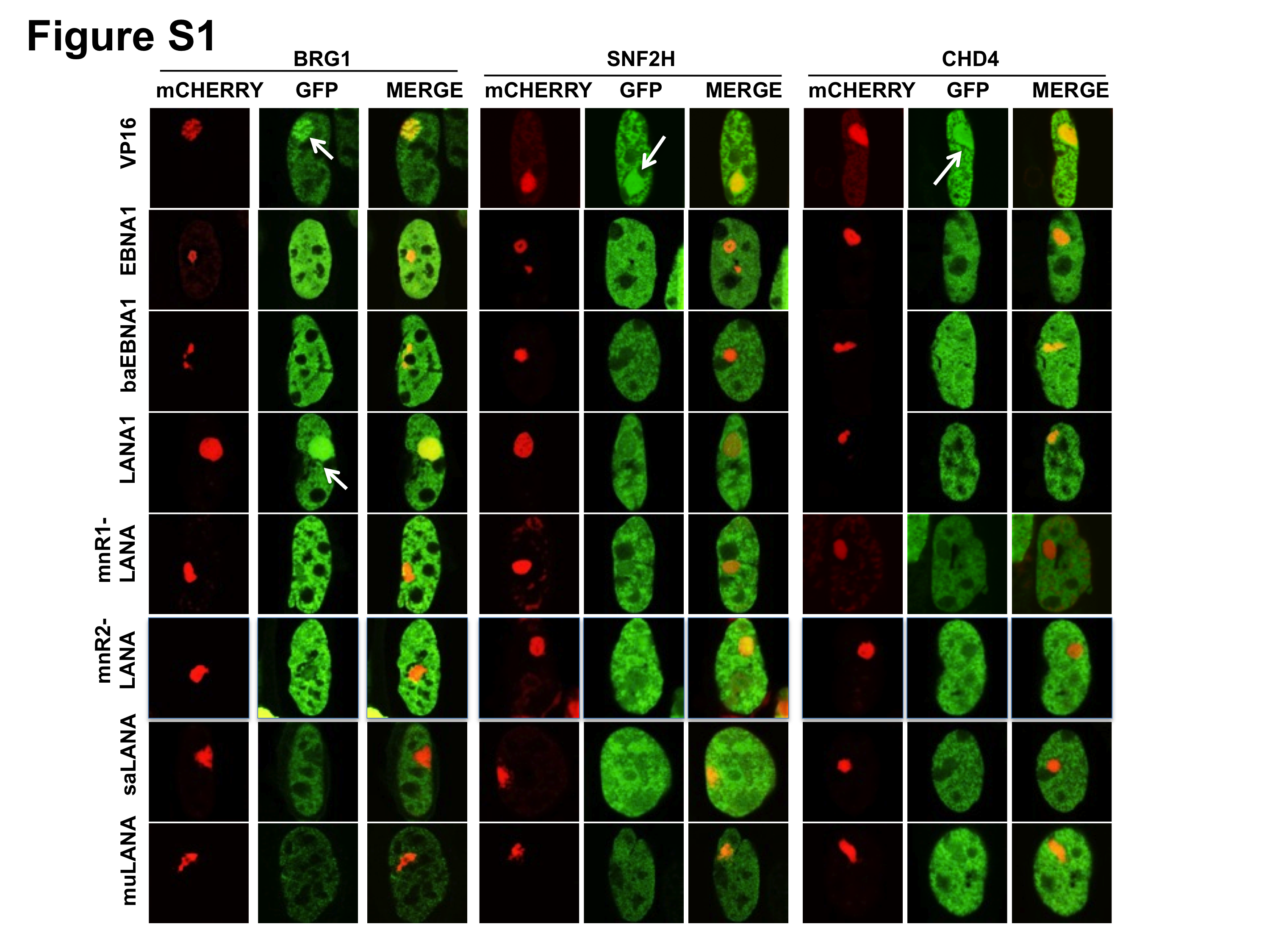

Supplement: Figure S1 — Recruitment of components of chromatin remodeling complexes by LCV and RHV GMPs. Representative confocal images illustrating the nuclear fluorescence of A03-1 cells co-transfected with plasmids expressing the mCherry-LacR-VP16 or mCherry-LacR-GMP fusion proteins and the GFP-tagged ATPase subunits of the SWI/SNF, ISWI and CHD4 chromatin remodeling complexes, BRG1, SNF2H and CHD4, respectively. Recruitment to the site of chromatin decondensation, visualized by the accumulation of green fluorescence overlapping with the red fluorescent array, is indicated by arrows. (TIFF) [file pone.0062783.s001.tiff]

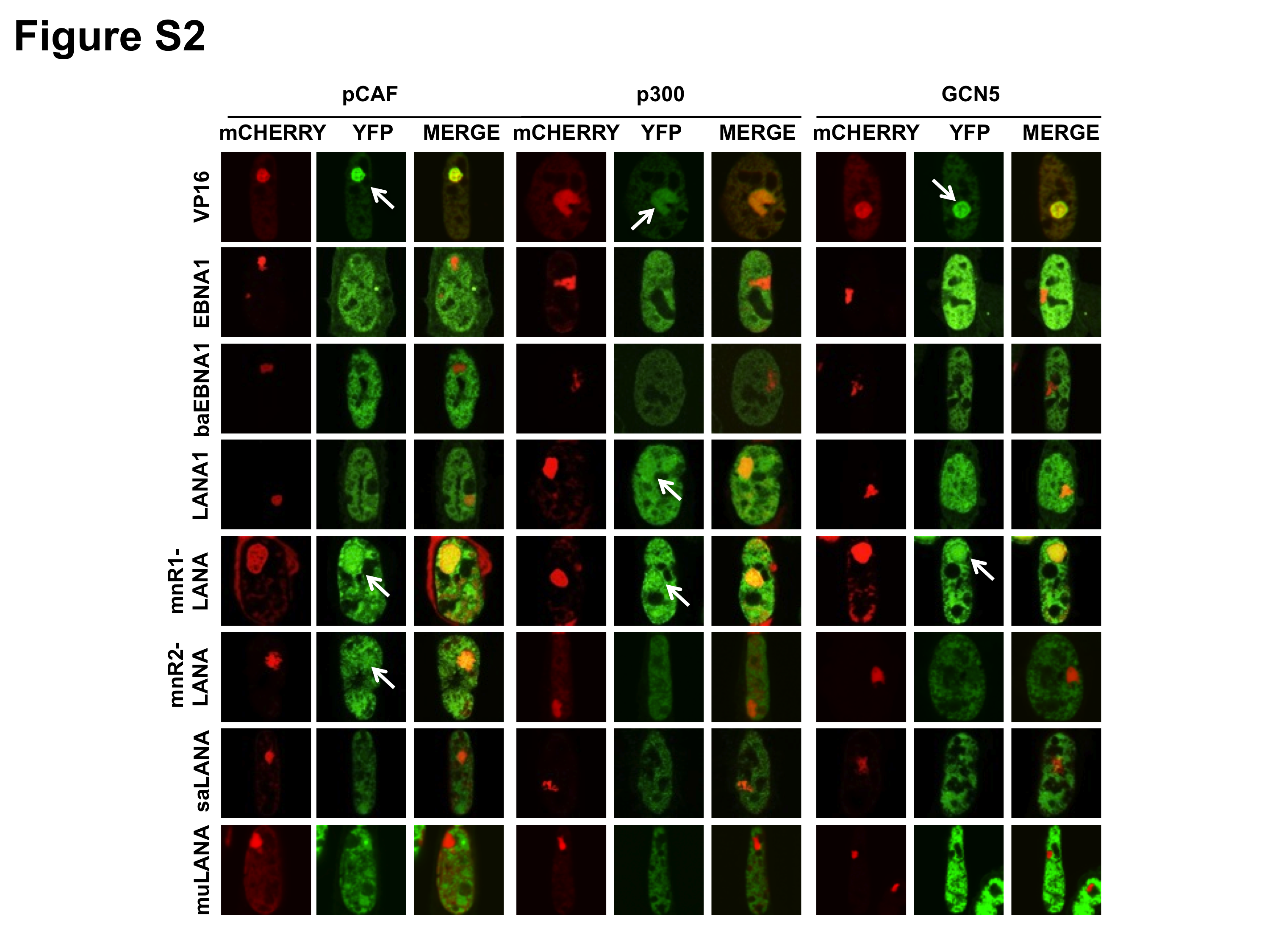

Supplement: Figure S2 — Recruitment of components of chromatin remodeling complexes by LCV and RHV GMPs. Representative confocal images illustrating the nuclear fluorescence of A03-1 cells co-transfected with plasmids expressing the mCherry-LacR-VP16 or mCherry-LacR-GMP fusion proteins and the YFP-tagged acetyltransferases pCAF, p300 and GCN5. Recruitment to the site of chromatin decondensation, visualized by the accumulation of green fluorescence overlapping with the red fluorescent array, is indicated by arrows. (TIFF) [file pone.0062783.s002.tiff]
